# Moving beyond Technical Issues to Stakeholder Involvement: Key Areas for Consideration in the Development of Human-Centred and Trusted AI in Healthcare

**DOI:** 10.1007/s41649-024-00300-w

**Published:** 2024-06-21

**Authors:** Jane Kaye, Nisha Shah, Atsushi Kogetsu, Sarah Coy, Amelia Katirai, Machie Kuroda, Yan Li, Kazuto Kato, Beverley Anne Yamamoto

**Affiliations:** 1 Centre for Health, Law, and Emerging Technologies (HeLEX), Faculty of Law, University of Oxford, Oxford, UK; 2 Melbourne Law School, University of Melbourne, Melbourne, VIC Australia; 3 Department of Biomedical Ethics and Public Policy, Graduate School of Medicine, Osaka University, Osaka, Japan; 4 Research Center on Ethical, Legal, and Social Issues, Osaka University, Osaka, Japan; 5 Center for Global Initiatives, Osaka University, Osaka, Japan; 6 Graduate School of Human Sciences, Osaka University, Osaka, Japan

**Keywords:** AI, Health, Governance, Human-centred, Trusted AI, Technology

## Abstract

Discussion around the increasing use of AI in healthcare tends to focus on the technical aspects of the technology rather than the socio-technical issues associated with implementation. In this paper, we argue for the development of a sustained societal dialogue between stakeholders around the use of AI in healthcare. We contend that a more human-centred approach to AI implementation in healthcare is needed which is inclusive of the views of a range of stakeholders. We identify four key areas to support stakeholder involvement that would enhance the development, implementation, and evaluation of AI in healthcare leading to greater levels of trust. These are as follows: (1) aligning AI development practices with social values, (2) appropriate and proportionate involvement of stakeholders, (3) understanding the importance of building trust in AI, (4) embedding stakeholder-driven governance to support these activities.

## Introduction 

Artificial intelligence (AI) systems are starting to have a profound effect on healthcare delivery and outcomes. It recognised that AI is not only reconfiguring relationships between healthcare professionals and patients (Topol 2019a, 2019b; Adams et al. 2020; Yang et al. 2019; Young et al. 2021) but also the relationships between clinical professionals and the way clinical practice is approached. Most significantly, AI is transforming the way in which patients, themselves, engage in health promotion, disease prevention, and monitoring activities (Feng et al. 2021). We need to understand better how to work with, and fully integrate, this new reality in healthcare practice (Pai et al. 2014). The potential scope and scale of the implications of AI in healthcare, therefore, requires a full understanding not just of the technology, but also the complex socio-technical issues associated with successful implementation.

Currently, studies of “medical AI has been largely technology-centric with a focus on the capabilities and accuracy of AI models” (Andersen et. al. 2023). Alongside the technical focus, there has been considerable attention on the development of ethical principles to act as guardrails to guide the development of AI technologies. This consists of general international documents (Corrêa et al. 2023) as well as the more specific guidance for health provided by the World Health Organisation (WHO 2021, 2024). However, we would argue that this is insufficient and more attention needs to be given to the socio-technical issues around the implementation of AI that are the key concerns of stakeholders—healthcare professionals, patients, members of the public, and the clinical researchers involved in the development and implementation of AI technologies in healthcare. We contend that a human-centred approach is essential to develop AI systems that “operate in ways that support and engender, for example, trust, fairness, or human control” (Andersen et al. 2023). Central to a human-centred approach is stakeholder engagement and involvement. This should be regarded as a critical element of the governance structure that supports the development of AI in a healthcare setting.

We have identified four key considerations for meaningful stakeholder involvement and the development of a more human-centred approach to the implementation of AI. This would encourage a greater alignment of AI technology development with human and societal values (Andersen et al. 2023). This could lead, hopefully, to the enhanced introduction of AI into clinical practice by facilitating enhanced understanding between all stakeholders leading to greater acceptance and trust of the use of AI in healthcare. These four considerations are as follows: (1) aligning AI development practices with social values, (2) appropriate and proportionate involvement of stakeholders, (3) understanding the importance of building trust in AI, (4) embedding stakeholder-driven governance. In this short perspective paper, we outline each of these points in more detail with the aim of furthering the discussion around the implementation of stakeholder involvement as a key requirement as AI technologies become integrated into healthcare.

## Aligning AI Development Practices with Social Values

The rapid innovation of AI in healthcare has created little time to meaningfully investigate the impact of these technologies on patients and healthcare professionals. The pressure of implementation schedules and the way that innovation processes are focused on the technical challenges can often discourage reflection on the social impact of AI systems and how these need to be fully understood for the successful adoption of AI technology. For AI to work for all in healthcare, an “AI for good” mindset and approach assists in prioritising patient safety and minimising bias and inequalities. According to recent reports, these are protective values that developers and adopters of AI should underscore in AI design and development over and above profit-making values (WHO 2021; UNESCO 2022). The former are often major concerns for patients, public, and healthcare professionals about AI in healthcare (Banerjee et al. 2022; Richardson et al. 2021).

Key to bringing social values into AI decision-making is understanding what the concerns of stakeholders are, particularly those of patients who are at the heart of healthcare systems. Although there is a rich history of patient and public involvement (PPI) in healthcare, particularly in the UK, this approach has not been adopted in the context of modern AI (Banerjee et al. 2022). Indeed, a criticism of the way in which AI systems and the practices surrounding them have been developed to date is that those who will be most impacted by AI, such as patients, are rarely (and some groups never) engaged with through dialogue nor are they put at the centre of decision-making.

This is contrary to WHO Guidance on the Ethics and Governance of Artificial Intelligence that recommends that important decisions about healthcare are made not only by civil servants and industry actors, but also through public participation of a wide range of potential users and all direct and indirect stakeholders (WHO 2021, 2024). While the ethical landscape surrounding AI is becoming well defined, governance frameworks are currently insufficient. To enable healthcare to benefit from the implementation of AI, professionals, patients, and the general public need to be confident that AI systems are safe and secure, and that processes are in place to mitigate against risks as well as legal and ethical concerns. This requires mechanisms that enable meaningful consideration of a wide range of stakeholder needs and concerns, and as well as clarity about when to involve them in decision-making across the development, implementation, and evaluation phases.

Exclusion from decision-making is a significant issue for people from diverse backgrounds in both Japan and the UK, where they are generally underserved and underrepresented in healthcare. The well-recognised danger with AI is that it can replicate and amplify the way that excluded groups are treated. Leaving the underserved out of decision-making can entrench epistemic exclusion, reaffirming negative stereotypes and discriminatory practices. The use of AI technology provides the opportunity to do things differently. Part of this is regarding patients as team members integral to successful technology development and adoption, rather than as people who have things done to them. A move towards the inclusion and participation of a wide range of patient’s voices in the development of AI technologies is likely to greatly improve the design of the technology and potentially lead to optimal adoption and implementation.

It is not only patients who are affected by the focus on the technical capabilities and accuracy of AI models rather than the socio-technical issues. In cases where there has been consultation with healthcare professionals, there can be significant differences in who, when, and how people are consulted. For example, in one study it was found that clinicians were consulted “at various but inconsistent points during the design process, and most typically at later stages in the design cycle (82%, 19/24 design studies)” (Tulk Jesso et al. 2022). If the notion that AI should be for all and used for good is adhered to, then a proactive approach is needed to improve acceptance and confidence among users and those impacted. Therefore, at a fundamental level, ways to incorporate stakeholder expectations and concerns should be made to inform decisions surrounding AI systems.

It is evident that the development and adoption of AI are not uniform, with considerable differences within countries, but also between countries (Ali et al. 2023). We know from our own experiences in Japan and the UK that there are “islands of innovation,” where particular hospitals or universities might be excelling in AI implementation, but that is not uniform throughout the country. At this early stage of development and implementation of AI in healthcare, it is inevitable that we will see a concentration in institutions of excellence. However, in addition, the digital infrastructure of health systems can be fragmented, with decisions about the provision of healthcare often being decided by the level of local resources. It is important to understand how these issues interact with individual and group factors or characteristics, as they have the potential to increase the inequalities associated with access to healthcare. This requires more than simply interacting with stakeholders and involving them in the development and adoption of AI. There is a need to not only understand the concerns of individuals but also the wider systemic factors that may lead to inequitable access to safe AI-powered or assisted healthcare. Understanding and addressing these bigger concerns makes clear the need for digital maturity among healthcare providers (Duncan et al. 2022) which in turn requires the consideration of the social impact of AI in different settings and the readiness for healthcare systems to mitigate risks and broaden the benefits of AI development and implementation for marginalised groups.

## Appropriate and Proportionate Involvement of Stakeholders

While stakeholder inclusion is important, we contend that it should be carried out only with clearly articulated aims and employ engagement and involvement methods that are appropriate and proportionate to the task. Identifying who to include, when, and for what purpose should form part of any AI development and implementation plans in healthcare. Not every stakeholder will need to be, or want to be, involved in every decision, nor should they all be involved in all the decision-making along the translational pathway. For engagement and involvement to be appropriate, it must be suitable for the stakeholder to carry out the task, as they are directly affected or have (or after training be able to acquire) the requisite knowledge and authority to make judgments about the issues of concern. It must also be proportionate, so that individuals are not asked to do things that are burdensome, and that the involvement does not create duplication or unnecessary oversight but adds value to the AI development and translational pathway. Therefore, an assessment must be made about how, and when, to involve different stakeholders and what methods should be used.

To begin aligning social values with technical development and implementation, the question of how involvement and engagement should be approached depends on the socio-technical context, the needs of the AI project (Banerjee et al. 2022), and the type of AI being used. The key questions all projects should ask are as follows: who needs to be involved for each decision and for which step of an AI’s lifecycle and how underrepresented groups can be meaningfully included in each relevant decision. Stakeholders and those impacted may also be engaged or involved in either passive or active ways. Both approaches have different purposes. For example, passive engagement can inform and raise awareness about uses of AI or the ethical and social impact issues, whereas active ways would try to capture attitudes and concerns of stakeholders, and a higher level of participation would involve the co-design and coproduction of activities and outputs (Banerjee et al. 2022).

The potential of digital tools to be used for public engagement and involvement should be appropriate and proportionate in the healthcare context where AI is being implemented. Engagement and involvement can be synchronous, or can be spread out and be asynchronous, so that different types of groups are included in a timely manner for different purposes. For example, messaging platforms allow many different people to be involved in debates and online meeting software reduces the physical and time burden involved in face-to-face meetings (Hamakawa et al. 2021; Kogetsu and Kato 2022). Interactive Dynamic Consent platforms can enable ongoing interaction and communication with patients and research participants over time according to the needs of the study (Schuler Scott 2022; Kaye et al. 2015).

However, once again, we advocate that this needs to be proportionate so that the intensity of engagement is appropriate, and that the best tools are used to facilitate this. For example, a relatively low intensity of engagement can be achieved by informative websites, interactive online Q&A sessions, or surveys. At a higher level of intensity, co-drafting position papers, discussion forums, and advocacy platforms focusing on AI in healthcare can be used to considerable effect both in real time and asynchronously to facilitate higher intensity engagement (Haas Lyons 2017; Hughes 2020). Again, there should be clear strategies to involve people from diverse backgrounds.

As AI capabilities evolve, societal expectations will also change, as will the pathways through which individuals navigate healthcare and relations between individuals and systems (between patients, clinicians, other professionals, and digital systems). This requires a constant review of engagement and involvement strategies to make sure that the methodologies used are still fit for purpose. Alongside this, it is crucial that there is a review of the type and amount of information and the way that it is presented, so that it continues to be appropriate for the context and the stakeholders involved (Theodorou et al. 2017).

## Understanding the Importance of Building Trust in AI

Researchers and engineers aim to ensure the reliability, efficacy, efficiency, and therefore trustworthiness of the AI technologies they develop. This is a fundamental starting point of technical design, but trustworthiness by such definitions does not mean that all stakeholders will trust AI healthcare technologies. Trust is a product of previous, current, and future relationships of different stakeholders with the healthcare system; trust in the use of AI in healthcare will be determined according to the reliability, competence, and intentions enacted by potential applications and implementation environments (Starke et al 2022). Crucial to building trust is establishing good relationships between patients and healthcare professionals and delivering high quality patient care. If patients trust healthcare professionals and the healthcare system, they will be more likely to trust AI technology. Where trust in the healthcare system is low, the task of gaining trust in AI is even more demanding.

It is important that AI technologies are fit for purpose, meaning that the technology can achieve the expectations of patients and other users. With all the hype around AI, it is important for developers to recognise the limits of any new technology (Elish and Boyd 2018). Both healthcare professionals and patients need to have a clear idea of what can be achieved by implementing various AI technologies. This requires dialogue between developers and end users such as physicians and allied healthcare professionals. At the same time, it requires that there is sufficient time for frontline healthcare professionals to explain the expectations of using a certain technology.

In the clinical setting, people want to know more about AI from trustworthy sources. If they have a high level of trust and confidence in healthcare systems, they will want the strong protections that surround healthcare data and clinical processes to be extended to AI. This will allow trust to be placed with others to make the right decisions for healthcare and society. In addition, acknowledging transparency, access to information, and patient involvement in decision-making are key facets of trust in the healthcare context not only in the UK and Japan, but many other countries. In clinical practice, patients want to know that AI systems are implemented with the strong support and understanding of healthcare professionals. They want to be able to discuss concerns with them, and for there to always be a “human in the loop” (Middleton et al. 2022) checking AI data inputs and outputs as well as clinical recommendations.

This is reflective of the current status of AI implementation, whereby systems are predominantly presented as tools to assist physicians and other healthcare professionals. AI systems are expected to evolve over time to become a routine part of healthcare that will engender confidence in decision-making. In the interim, there is concern that the assistance of AI may erode clinical autonomy and deskill those making clinical decisions. There is also concern that AI systems might nudge, or encourage, clinicians to make decisions that would not otherwise be made, due to a lack of transparency or explainability around how decisions have been made (Katirai et al. 2023). There are also concerns that AI will reduce patient autonomy and decision-making and erode the trust that currently exists between patients and healthcare professionals. To fully understand the complexities of maintaining trust in a healthcare system that implements AI requires constant stakeholder involvement to understand the issues associated with the technology as these change over time.

## Embedding Stakeholder-Driven Governance

Considerable efforts have been made to develop ethical principles, legislation, guidelines, and standards (WHO 2021, 2024; UNESCO 2022) to scaffold the effective and ethical development of AI in healthcare. Although there are strong recommendations for stakeholder involvement and engagement in many influential reports (WHO 2021; UNESCO 2022), this is not routinely put into practice. It does not help that such requirements are not enforced in law by nation-states. Therefore, an entirely different level of approach is needed to make AI use more human-centred and for this to be enforceable and accountable.

One suggestion that does not require the passing of legislation is the development of a social charter at a national level in each country dealing specifically with AI usage in healthcare, in the similar way to the Patient and the Health Professional Responsible for Care (HCP) Constitutions of the National Health Service in the UK (Department of Health and Social Care 2023). Developing a dedicated social charter would ensure the relevant stakeholders share the benefits as well as responsibilities of these technologies. A possible charter would outline the rights and responsibilities of both patients and healthcare professionals as well as incorporate pledges that would help to maintain standards. They would clarify the requirements for more open and stakeholder-driven governance and what that would look like in practice.

Such a charter should be a “living” document co-designed with all local stakeholders impacted by changes to current healthcare delivery practices, enabling the transition to intelligent healthcare to be better aligned with the needs and desires of the wider society. In this way, it could reflect local concerns and interests. While stakeholder preferences will always be nuanced by local contexts, a national charter would also need to recognise that the technological revolution powered by AI is also occurring on a global stage. It would have to reflect an awareness of differences in the interpretations of human rights between regions and avoid increasing existing disparities between high- and low-income countries. It should take into account the fact that AI solutions are predominantly created with data derived from highly developed countries but will be implemented in local contexts around the globe (Shaw et al. 2024).

Furthermore, rules nuanced to more local contexts are necessary. AI implementation brings influence on various levels—from an individual level to a society level. It will result in fundamental change of behaviours of patients and healthcare professionals, the nature of medical practice, and medical systems. To avoid confusion or antipathy as a result of these changes, it is necessary to regularly update standards, guidelines, and laws for medical practice that reflect the local context. These should be developed reflecting stakeholders’ values, perspectives, expectations, and concerns and be known and understood by all. At the same time, it is important to conduct holistic assessments of how AI impacts issues of professionalism, professional skills, evidence-based best practice, and relationships between patients and professionals. Given the rapid pace of change, it is hard to predict fully how each of these areas will be impacted, hence the need for regular assessments that take into account different stakeholders’ perspectives. This potentially could lead to improvements not only in the use of AI in healthcare, but also in healthcare itself.

## Conclusion

For those who are at the cutting-edge of technological development, there is an understandable focus on whether the technology works or not, that is: is it trustworthy? However, when taking a broader societal approach, we need to consider the necessary elements that will lead to AI systems being “trusted.” To address this question, we need to pay more attention to a variety of human factors. We need to understand the concerns and expectations of different stakeholder groups about the use of AI in healthcare. As Banerjee et al. opine “In order to build trust in AI algorithms, one needs to consider the complex socio-technological milieu in which technological solutions reside. Trust needs to be built not only in AI algorithms, but the training data, software, and complex environment in which humans are situated.” (Banerjee et al. 2022). Those with more input in decision-making or those who feel they have been consulted and kept informed may feel more inclined to trust the use of AI in healthcare (Steerling et al. 2023).

In short, what we are arguing here is that we need to make AI systems more human-centred. In particular, we need to take special care to involve people who may not normally be consulted but have a stake in the systems introduced. This applies as much to healthcare professionals as other stakeholder groups, such as patients and the public. Delineating which stakeholders will be informed, engaged, or involved and how that should occur in an appropriate and proportionate way should be part of early planning. Where needed, actively recruiting under-represented groups for direct involvement in development and implementation is important. Planning and delivering feedback to all stakeholders in the interests of transparency should also be part of the involvement process.

It should be stressed that what we are arguing for in this paper is not normal practice and will not organically occur. It requires commitment and action. We need to create strategies and build capacity for stakeholder inclusion within AI projects, that will ensure stakeholder concerns and expectations underpin, and drive activities and outputs. This requires investment to understand the socio-technical issues associated with implementation as well as the technological aspects of AI development. Given the huge investments in the field overall, we need some priority funding created as a part of all projects for the kind of stakeholder involvement suggested in this paper.

Establishing sustainable stakeholder platforms may reduce the burden in this endeavour. The creation of a platform, virtual or physical, that supports various engagement and involvement activities and brings together key stakeholders interested in contributing and engaging in different local settings will enhance recruitment and help build capacity. However, those who volunteer via a platform to engage with those rolling out different types of healthcare AI will represent only a few. There will always be a need for outreach work and the investment of time to ensure broader representation.

Creating spaces for inclusive dialogue with a broad range of stakeholders will address and re-centre our attention to issues of trust, safety, benefits, potential harm, security, and impact on agency. This could aid in the pursuit of equitable decision-making and the ethical underpinning of AI development and adoption. This will tip the balance away from focusing on making better algorithmic models to understanding the issues associated with implementation.

In this paper, we have identified four key areas to support stakeholder involvement that would enhance the development, implementation, and evaluation of AI in healthcare leading to greater trust. To move towards a more human-centred approach, two things need to happen. There is a need for a leadership to ensure that national regulations, governance structures, hospital guidelines, etc. are underpinned by a stakeholder involvement approach and, where necessary, financial support needs to be provided for this to be achieved. The other is recognition and identification of the stakeholders who should be involved, including various healthcare professionals as well as patients and citizens. This involvement should not be carried out in a way that is tokenistic (Joyce et al. 2021). The collective efforts by all relevant people will result in a more human-centred AI and the possibility that the technology is trusted by those in the wider society and by future generations.

The expected outcomes from identifying stakeholders’ preferences for AI use in healthcare and establishing shared decision pathways will help to align common goals and harmonise ways of working that achieve social benefits. Over and above this, clear mechanisms through safety-orientated and human-centred governance would generate trust and confidence in AI systems and create a benchmark for good practice and sustainable AI adoption.

## References

[CR1] Adams Scott J, Tang Rachel, Babyn Paul (2020). Patient perspectives and priorities regarding artificial intelligence in radiology: Opportunities for patient-centered radiology. Journal of the American College of Radiology.

[CR2] Ali Omar, Abdelbaki Wiem, Shrestha Anup, Elbasi Ersin, Alryalat Mohammad Abdallah Ali, Dwivedi Yogesh K (2023). A systematic literature review of artificial intelligence in the healthcare sector: Benefits, challenges, methodologies, and functionalities. Journal of Innovation & Knowledge.

[CR3] Andersen Tariq Osman, Nunes Francisco, Wilcox Lauren, Coiera Enrico, Rogers Yvonne (2023). Introduction to the special issue on human-centred AI in healthcare: Challenges appearing in the wild. ACM Transactions on Computer-Human Interaction.

[CR4] Banerjee Soumya, Alsop Phil, Jones Linda, Cardinal Rudolf N (2022). Patient and public involvement to build trust in artificial intelligence: A framework, tools, and case studies. Patterns.

[CR5] Department of Health and Social Care. 2023. NHS constitution for England. https://www.gov.uk/government/publications/the-nhs-constitution-for-england/the-nhs-constitution-for-england. Accessed 8 Nov 2023.

[CR6] Duncan Rhona, Eden Rebekah, Woods Leanna, Wong Ides, Sullivan Clair (2022). Synthesizing dimensions of digital maturity in hospitals: Systematic review. Journal of Medical Internet Research.

[CR7] Elish Madeleine Clare, Boyd Danah (2018). Situating Methods in the magic of big data and AI. Communication Monographs.

[CR8] Feng Shan, Mäntymäki Matti, Dhir Amandeep, Salmela Hannu (2021). How self-tracking and the quantified self promote health and well-being: Systematic review. Journal of Medical Internet Research.

[CR9] Haas Lyons, Susanna. 2017. Digital engagement, social media & public participation. *International Association for Public Participation*. https://www.iap2canada.ca/resources/Documents/Newsletter/2017_social_media_white_paper.pdf. Accessed 8 Nov 2023.

[CR10] Hamakawa Nao, Kogetsu Atsushi, Isono Moeko, Yamasaki Chisato, Manabe Shirou, Takeda Toshihiro, Iwamoto Kazumasa (2021). The practice of active patient involvement in rare disease research using ICT: Experiences and lessons from the RUDY JAPAN project. Research Involvement and Engagement.

[CR11] Hughes Ashley M (2020). Artificial intelligence-enabled healthcare delivery and real-time medical data analytics in monitoring, detection, and prevention of COVID-19. American Journal of Medical Research.

[CR12] Joyce, Kelly, Laurel Smith-Doerr, Sharla Alegria, Susan Bell, Taylor Cruz, Steve G. Hoffman, Safiya Umoja Noble, and Benjamin Shestakofsky. 2021. Toward a sociology of artificial intelligence: A call for research on inequalities and structural change. *Socius* 7: 237802312199958. 10.1177/2378023121999581.

[CR13] Katirai Amelia, Yamamoto Beverley Anne, Kogetsu Atsushi, Kato Kazuto (2023). Perspectives on artificial intelligence in healthcare from a patient and public involvement panel in japan: An exploratory study. Frontiers in Digital Health.

[CR14] Kaye Jane, Whitley Edgar A, Lund David, Morrison Michael, Teare Harriet, Melham Karen (2015). Dynamic consent: A patient interface for twenty-first century research networks. European Journal of Human Genetics.

[CR15] Kluge Corrêa Nicholas, Galvão Camila, Santos James William, Del Pino Carolina, Pinto Edson Pontes, Barbosa Camila, Massmann Diogo, Mambrini Rodrigo, Galvão Luiza, Terem Edmund, de Oliveira Nythamar (2023). Worldwide AI ethics: A review of 200 guidelines and recommendations for AI governance. Patterns.

[CR16] Kogetsu Atsushi, Kato Kazuto (2022). Framework and practical guidance for the ethical use of electronic methods for communication with participants in medical research. Journal of Medical Internet Research.

[CR17] Middleton Stuart E, Letouzé Emmanuel, Hossaini Ali, Chapman Adriane (2022). Trust, regulation, and human-in-the-loop AI: Within the European region. Communications of the ACM.

[CR18] Pai Vinay M, Rodgers Mary, Conroy Richard, Luo James, Zhou Ruixia, Seto Belinda (2014). Workshop on using natural language processing applications for enhancing clinical decision making: An executive summary. Journal of the American Medical Informatics Association.

[CR19] Richardson Jordan P, Smith Cambray, Curtis Susan, Watson Sara, Zhu Xuan, Barry Barbara, Sharp Richard R (2021). Patient apprehensions about the use of artificial intelligence in healthcare. Npj Digital Medicine.

[CR20] Schuler Scott, Arianna. 2022. Dynamic consent: A mechanism for engagement. PhD diss., University of Oxford. https://ora.ox.ac.uk/objects/uuid:a671615d-ebed-4a7e-987a-481167b1056f. Accessed 10 June 2024.

[CR21] Shaw James, Ali Joseph, Atuire  Caesar A, Cheah  Phaik Yeong, Guio Español Armando, Gichoya Judy, Hunt Adrienne, Jjingo Daudi, Littler  Katherine, Paolotti  Daniela, Vayena Effy (2024). Research ethics and artificial intelligence for global health: Perspectives from the global forum on bioethics in research. BMC Medical Ethics.

[CR22] Starke Georg, van den Brule Rik, Elger  Bernice Simone, Haselager Pim (2022). Intentional machines: A defence of trust in medical artificial intelligence. Bioethics.

[CR23] Steerling Emilie, Siira Elin, Nilsen Per, Svedberg Petra, Nygren Jens (2023). Implementing AI in healthcare—the relevance of trust: A scoping review. Frontiers in Health Services.

[CR24] Theodorou Andreas, Wortham Robert H, Bryson Joanna J (2017). Designing and implementing transparency for real time inspection of autonomous robots. Connection Science.

[CR25] Topol Eric J (2019). Deep medicine: How artificial intelligence can make healthcare human again.

[CR26] Topol Eric J (2019). High-performance medicine: The convergence of human and artificial intelligence. Nature Medicine.

[CR27] Tulk Jesso, Stephanie, Aisling Kelliher, Harsh Sanghavi, Thomas Martin, and Sarah Henrickson Parker. 2022. Inclusion of clinicians in the development and evaluation of clinical artificial intelligence tools: A systematic literature review. *Frontiers in Psychology* 13: 830345. 10.3389/fpsyg.2022.830345. 10.3389/fpsyg.2022.830345 PMC9022040 35465567

[CR28] UNESCO. 2022. Recommendation on the ethics of artificial intelligence. https://unesdoc.unesco.org/ark:/48223/pf0000381137. Accessed 8 Nov 2023.

[CR29] WHO. 2021. Ethics and governance of artificial intelligence for health. *World Health Organisation*, 28 June 2021. https://www.who.int/publications/i/item/9789240029200. Accessed 8 Nov 2023.

[CR30] WHO. 2024. Ethics and governance of artificial intelligence for health: Guidance on large multi-modal models. *World Health Organisation*, 18 January 2024. https://www.who.int/publications/i/item/9789240084759. Accessed 25 Apr 2024.

[CR31] Yang Keyi, Zeng Zhi, Peng Hu, Jiang Yu (2019). Attitudes Of Chinese cancer patients toward the clinical use of artificial intelligence. Patient Preference and Adherence.

[CR32] Young Albert T, Amara Dominic, Bhattacharya Abhishek, Wei Maria L (2021). Patient and general public attitudes towards clinical artificial intelligence: A mixed methods systematic review. Lancet Digital Health.

